# The role of universities in society

**DOI:** 10.1126/sciadv.adx2929

**Published:** 2025-11-07

**Authors:** Michèle Belot, Lea Cassar, Karoline Ströhlein

**Affiliations:** ^1^ Department of Economics, Cornell University, Ithaca, NY 14853-3201, USA.; ^2^ Department of Economics and Econometrics, University of Regensburg, Regensburg 93053, Germany.

## Abstract

This study examines public views on universities’ societal roles beyond education and research. A representative sample of 2089 U.S. citizens evaluates whether universities should engage in initiatives such as diversity, equity, and inclusion (DEI), free speech, and environmental sustainability, benchmarking views against corporations. We include an incentivized experiment asking respondents to allocate money between universities based on performance on various dimensions. Results show broad support for engagement in initiatives related to environmental sustainability, well-being, and free speech but opposition to political engagement. Views on DEI are polarized: Women and liberals reward universities excelling in DEI but conservatives and men penalize them, even when academic performance is equal. These findings highlight the polarization surrounding universities’ societal roles.

## Teaser

Americans agree universities have a broader role beyond education and research but do not fully agree on what that role is.

## INTRODUCTION

Universities have increasingly become focal points of public debate about their responsibilities in moderating speech, fostering inclusivity, and engaging with politically sensitive issues (*1*–*4*). Recent events involving the most prestigious institutions in the country have revived long-standing questions about the role universities should play in society and the extent of their responsibilities beyond education and research.

In environments where a large share of the population studies or has studied (in the U.S., 38% of adults hold a bachelor’s degree or higher) (*5*), universities are well positioned to influence social attitudes. Historically, they have served as anchors for social change, from advancing civil rights to driving environmental movements (*6*–*10*). Whether and how they should engage in such roles has been a topic of debate for a long time (*11*, *12*).

This study provides insights into how the American public perceives the role of major universities, focusing on their involvement in initiatives that extend beyond their core mission of education and research, such as promoting environmental sustainability, diversity, equity, and inclusion (DEI), political engagement, and freedom of speech. We are interested in benchmarking these expectations relative to those for major for-profit corporations that also have potentially relevant societal and political influence, often engaging in initiatives that extend beyond their core business (*13*–*16*). We also examine how expectations about these roles vary across political and sociodemographic lines (men/women, conservatives/liberals, and college educated/non–college educated).

We report evidence from a large online survey with an embedded incentivized experiment involving 2089 American citizens, representative of the population in age, gender, and political affiliation. The survey was conducted in December 2024, after President Trump had been elected, but before he took office and before he signed executive orders targeting universities. The responses to our survey demonstrate broad public support for universities and corporations engaging in socially relevant initiatives beyond their core missions, such as initiatives related to environmental sustainability, DEI, and free speech. However, initiatives related to political engagement are perceived as inappropriate. While these views are broadly shared across gender and education groups, a clear political divide emerges, particularly regarding the role of universities. The main points of disagreement involve political engagement and DEI, which are seen as desirable by liberals but are opposed by conservatives, and patriotism, for which the opposite is true.

Polarization over the role of universities appears even more clearly in an incentivized allocation experiment, where respondents were asked to allocate money ($30) between two anonymous universities, based on their rank in four specific dimensions: academic performance, environmental sustainability, DEI, and freedom of speech. Women and liberals consistently reward universities with better rankings across all dimensions, allocating more money accordingly. In contrast, men and conservatives penalize universities with better performance in DEI, allocating them less money all else being equal.

## RESULTS

### Views on the role of universities versus corporations

We begin by examining expectations regarding the initiatives universities and corporations should engage in. Respondents were asked the following question: “In your opinion, should universities engage in the following initiatives beyond their core mission (which is to conduct cutting-edge research and educate students)” or “In your opinion, should corporations engage in the following initiatives beyond their core mission (which is to conduct their core business)”?

**Fig. 1.**
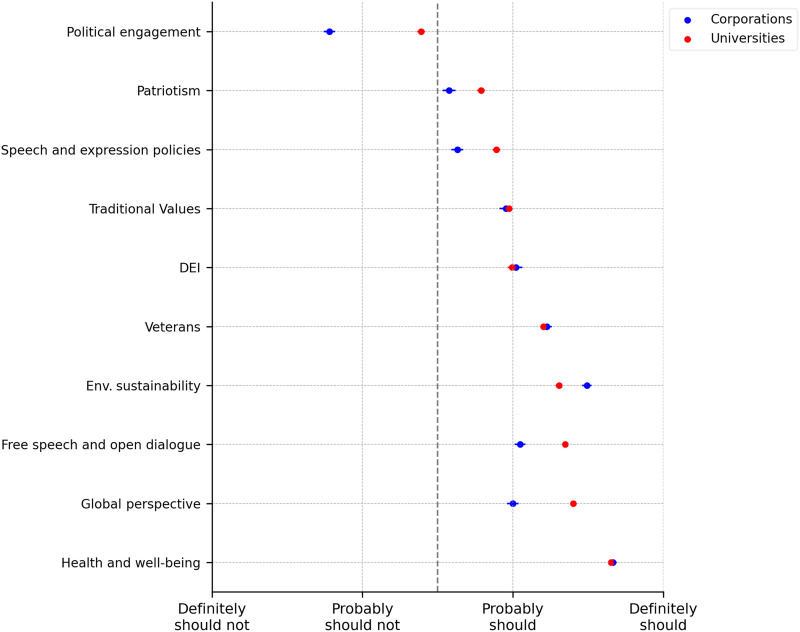
Initiatives universities/corporations should engage in. Dots indicate means, whiskers indicate ±1 SE. Results are based on answers to the question “In your opinion, should universities/corporations engage in the following initiatives beyond their core mission (which is to conduct cutting-edge research and educate students/which is to conduct their core business)?” with the following answer options: Definitely should engage in/probably should engage in/probably should not engage in/definitely should not engage in.

The initiatives listed included DEI, environmental sustainability, political engagement, speech and expression policies, traditional values, health and well-being programs, global perspectives, free speech and open dialogue, patriotism and veterans’ initiatives. The results are shown in Fig. 1, and the corresponding table with *P* values for *t* tests of equality of means for universities and corporations are shown in table S1. Overall, there appears to be broad public support for both universities and corporations to engage in initiatives beyond their core missions. Aside from political engagement, which is seen as inappropriate; the public is supportive of universities and corporations engaging in all the initiatives proposed. The most strongly supported initiatives are health and well-being programs, followed by initiatives related to fostering a “global perspective.” Liberal-leaning initiatives, such as environmental sustainability and DEI, also receive strong endorsement. In addition, more conservative initiatives, including free speech and open dialogue, traditional values, and prioritizing veterans, are viewed as appropriate for both universities and corporations. These findings show that the public is supportive of active engagement of universities in goals of broad societal relevance, and these expectations are similar to those for corporations. Given the very different missions and structures of these two types of institutions, this degree of similarity may be unexpected, suggesting perhaps that large institutions, regardless of sector, should contribute to societal goals.

While this general pattern of support holds across both institutional types, there are noticeable differences. Political engagement is viewed as inappropriate for both types of institutions, yet it is seen as less inappropriate for universities than for corporations (*t* test, P=0.000 ), suggesting that political expression is considered more acceptable in academic settings. Support is also somewhat higher for universities when it comes to initiatives related to patriotism, speech and expression policies, free speech, and global perspective (*t* tests, P=0.000 ). In contrast, environmental sustainability receives slightly stronger support in the corporate context (*t* test, P=0.000 ), perhaps because, although this remains speculative, corporations are seen as more directly responsible for environmental harm. Together, these patterns indicate that while public expectations for universities and corporations are broadly similar, they are not identical: With the exception of environmental sustainability, universities are expected to engage in a slightly broader range of societal initiatives.

### Sociodemographic differences in expectations

**Fig. 2.**
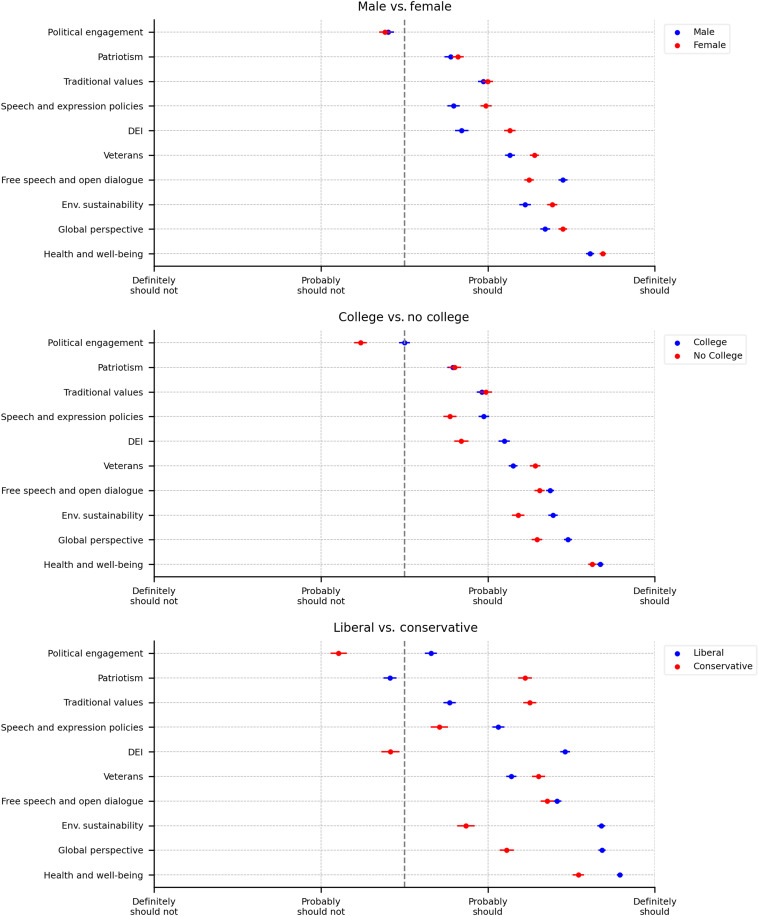
Initiatives universities should engage in, sociodemographic differences. Dots indicate means, whiskers indicate ±1 SE. Results are based on answers to the question “In your opinion, should universities engage in the following initiatives beyond their core mission (which is to conduct cutting-edge research and educate students)?” with the following answer options: Definitely should engage in/probably should engage in/probably should not engage in/definitely should not engage in.

These aggregate results could, however, mask heterogeneities across sociodemographic groups. Figure 2 presents the mean expectations for universities, with the sample split along three sociodemographic dimensions: gender (male versus female), education (college degree versus no college degree), and political orientation (conservative versus liberal). Tables S2 to S4 present the raw means plotted in the figure, as well as the *t* tests of equality across subgroups. Table S5 presents ordinary least squares (OLS) estimates of the combined role of these sociodemographic factors on the support for initiatives. This analysis includes controls for a wide range of sociodemographic characteristics, including age, presence of children, race, religion, employment status, occupation, industry, state of residence, and yearly income. It also accounts for a category of respondents that cannot be classified as conservatives or liberals based on their survey responses (classified as “other political”).

Overall, there is broad consensus across gender and education groups that universities should engage in most initiatives, apart from political engagement. A notable exception is respondents with a college degree who, on average, hold a neutral position, neither strongly in favor nor against universities engaging in political engagement. Women and college-educated individuals are generally more supportive of active involvement, in particular in the areas of speech and expression policies, DEI, and environmental sustainability (*t* tests, *P* = 0.000).

Views are more diverse and polarized along the political lines. The differences are largest in views about initiatives related to DEI and political engagement. DEI in particular is strongly supported by liberals while conservatives are more neutral (*t* tests, *P* = 0.000). Conversely, conservatives strongly support initiatives related to patriotism, while liberals are more neutral (*t* test, *P* = 0.000). Among initiatives that receive widespread support, liberals are more supportive of environmental sustainability and global perspectives, whereas conservatives favor initiatives related to traditional values and veterans. These differences reflect broader ideological divides observed in public attitudes toward policies and institutions (*17*). Despite these divisions, there is broad consensus that universities should actively support health and well-being initiatives, as well as free speech and open dialogue.

Comparing with the views on corporations, the degree of consensus is similar, although with a less pronounced political divide (see table S6). As with universities, liberals tend to favor greater corporate involvement in DEI (where conservatives, on average, hold a neutral stance), environmental sustainability, and global perspectives. In contrast, liberals oppose patriotism, which is strongly supported by conservatives, marking the primary point of disagreement. Overall, the results suggest that public expectations for corporations are more uniform, whereas universities are more likely to elicit more polarized views, perhaps because of the recent high-profile and politically divided debates over some of their actions.

Respondents were also asked to evaluate the current degree of engagement of universities and corporations with each of these initiatives. Their responses indicate that they find too little current engagement with the initiatives they wish universities or corporations engage with, and too much current engagement with those they wish they would not engage with. The results are presented in tables S7 and S8.

### Allocation task

We now turn to the allocation task experiment, where respondents allocated $30 between two universities (labeled A and B) ranked across four dimensions: academic performance, environmental sustainability, DEI, and free speech. The ranks were taken from publicly available sources (details are provided in Materials and Methods). Each respondent made five allocation decisions, selected from 22 possible combinations of universities’ pairs.

**Fig. 3.**
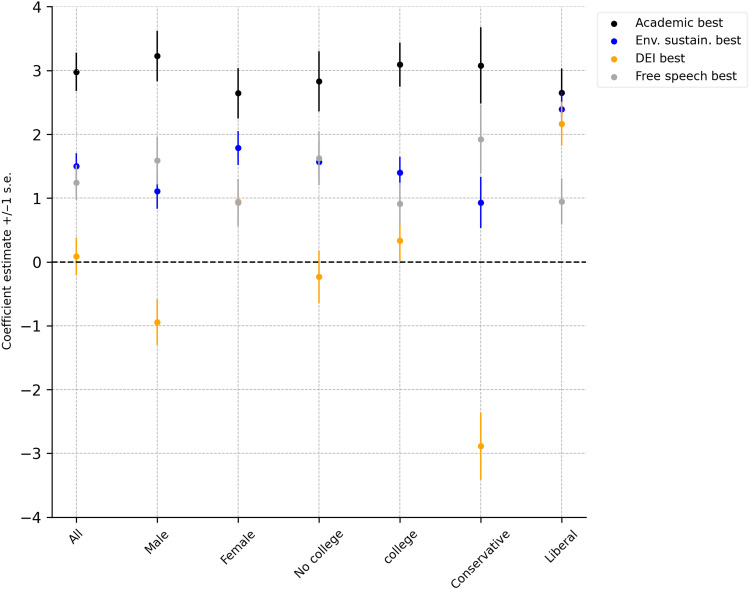
Difference in dollars allocated between universities. Dots indicate estimated OLS coefficients from the regressions reported in table S9, whiskers indicate ±1 SE. Dependent variable is the amount allocated to University A, the independent variables are dummies indicating if, relative to University B, A is ranking higher in academic performance, environmental sustainability, DEI, and free speech.

Figure 3 presents the estimated OLS coefficients from the regressions reported in table S9. The figure displays results for the full sample, as well as breakdowns by gender (men versus women), education (no college versus college), and political orientation (conservative versus liberal). The dependent variable is the amount allocated to University A. The key independent variables of interest are dummies indicating whether University A ranks higher than University B on academic performance, environmental sustainability, DEI, and free speech.

Academic performance stands out as the most influential factor in determining allocations. On average, respondents allocate $3 more (10% of the total) to universities ranking highest in academic performance, holding all else constant. The value placed on academic performance does not differ significantly across sociodemographic groups, indicating a shared prioritization of this dimension. However, differences emerge in its relative importance compared to other factors across the political spectrum: For conservatives, academic performance is not valued significantly more than free speech [ $\chi^{2}\left(1\right)=1.76$ and P=0.184 ], while for liberals, academic performance is as important as environmental sustainability [ $\chi^{2}\left(1\right)=0.28$ and  P=0.596] or DEI [ $\chi^{2}\left(1\right)=0.83$ and P=0.363].

Beyond academic performance, environmental sustainability and free speech emerge as the next most influential dimensions. While there is general agreement across groups regarding their importance, key differences arise: Liberals prioritize environmental sustainability over free speech, allocating 167% more to universities performing better in sustainability than to those ranking higher in free speech [$2.4 versus $0.9, $\chi^{2}\left(1\right)=11.38$ and P<0.001 ]. They also value sustainability significantly more than conservatives, who allocate only $0.9 more to universities performing better in that dimension, reflecting the same 167% difference [ $\chi^{2}\left(1\right)=9.08$ and P=0.003].

The most notable finding concerns the role of DEI rankings. On average, universities scoring higher on DEI do not receive more funding ( F=0.09 and P=0.762 ). However, this overall neutrality masks important differences across gender and political affiliation. Women and liberals allocate approximately $1 ( F=5.04 and P=0.025 ) and $2.2 ( F=26.74 and P=0.000 ) more, respectively, to universities with better DEI rankings. In contrast, men allocate $1 less to the higher-ranking DEI institutions ( F=5.10 and P=0.024 ), and conservatives allocate $2.9 less, approximately 10% of the total amount, toward these institutions ( F=21.81 and P=0.000 ). This suggests that men and conservatives are not only less supportive of DEI but actively penalize universities performing better in this dimension, even if academic performance is equal.

While the self-reported preferences and the allocation task yield broadly consistent results, the latter offers a clearer view of how the general public prioritizes different initiatives when trade-offs are required. Unlike the allocation task, which forces choices across multiple dimensions, the self-reports capture preferences along one dimension at a time. Some divergence is therefore expected, even under truthful reporting. Nevertheless, the self-reported preferences align well with observed allocation decisions. Respondents who are more supportive of the engagement of universities in a specific dimension do allocate on average more to the universities performing best in that dimension, although the correlations are relatively small: 0.24 ( P=0.000 ) for DEI, 0.05 ( P=0.000 ) for environmental sustainability, and 0.10 for free speech policies ( P=0.000 ). The ranking of initiatives in the survey is also consistent with the weights respondents attribute to the initiatives in the allocation task. For example, men indicate strongest support for initiatives related to free speech and the weight of free speech in the allocation task is the highest. We also find conservatives expressing the strongest support for free speech and sustainability and giving these two dimensions the strongest weight in the allocation task.

However, the results are not entirely consistent. For example, conservatives appear slightly against DEI initiatives in the survey, but the penalty they impose in the allocation experiment is substantial. Also, men are penalizing universities that score better in DEI in the allocation experiment, while they (weakly) support it in the survey. In our view, the design of the allocation task alleviates potential biases related to social image, experimenter demand effects, self-serving beliefs, or self-deception (*18*).

## DISCUSSION

The results from the survey demonstrate that Americans generally agree that universities have a broader role to serve in society, beyond education and research. The role they envision shares some similarities with the societal role envisioned for large for-profit corporations. There is broad support on average for a large set of initiatives, including initiatives related to health and well-being, environmental sustainability or veterans and traditional values. There is however a divide on whether universities should engage in DEI specifically, with women and liberals being in favor, while men and conservatives being against.

To gauge the robustness of our results, we conduct a series of additional analyses. First, we test alternative definitions of political affiliation. Our preregistered analysis was based on a variable defining political affinity based on responses to two questions: The first question is a question on political orientation on a scale from 1 to 7 where 1 means “strongly conservative” and 7 means “strongly liberal,” and political identity (Democrat/Republican/Independent/prefer not to answer). Respondents who answered the first question with 1 to 3 or the second with Republican were classified as conservative. Respondents who answered the first question with 5 to 7 or the second with Democrat were classified as liberal. If respondents were classified as both, conservative and liberal, they were dropped from the analysis (this was the case for 272 respondents). We gauge the robustness of the results when using alternative definitions of political affiliation, specifically, political identity and voting behavior (also preregistered). Political identity is based on the survey question where participants were asked if they identified as Democrat, Republican, Independent, or preferred not to answer. This classification yields a distribution closer to that of the General Social Survey, with 32% identifying as Democrats and 30% as Republicans. Results using this definition are reported in the Supplementary Materials: Tables S10 and S11 present the findings for survey responses, while the third and fourth columns of table S12 show results for the allocation task experiment. Across all analyses, the findings remain robust.

Voting behavior is based on self-reported voting behavior in the 2024 presidential election, categorizing respondents by whether they report having voted for Trump, Harris, or neither. The results are presented in tables S13 and S14 for survey responses and in the last two columns of table S12 for the allocation task experiment. While the overall patterns remain consistent under this alternative classification, a few differences emerge. In the allocation task, unlike conservatives [ $\chi^{2}\left(1\right)=6.14$ and P=0.013 ], Trump voters do not reward universities that score higher on environmental sustainability [ $\chi^{2}\left(1\right)=2.87$ and P=0.090 ]. Conversely, Harris voters reward universities with higher DEI scores even more than liberals do. That is, the disagreement on DEI is stronger between Trump and Harris voters than between liberals and conservatives. In contrast, Trump and Harris voters show greater alignment on the role of free speech [$1.5 versus $1.3, $\chi^{2}\left(1\right)=0.10$ and P=0.757 ], indicating more agreement on this issue than is observed between conservatives and liberals ($1.9 versus $0.9, $\chi^{2}\left(1\right)=2.19$ and P=0.139).

Additional secondary analyses and robustness checks are presented in the Supplementary Materials (see tables S15 to S18), following our preregistered plan. All main results remain consistent across various specifications and inclusion of controls. Note that the fraction of college-educated respondents in our sample (58%) exceeds the current national estimate (38%), likely due to our use of Prolific for participant recruitment, which tends to attract a more highly educated population. This over-representation should be kept in mind when considering the generalizability of our findings.

Last, we also report analyses of the support for engagement and the incentivized allocation task by race in the Supplementary Materials (tables S19 and S20). The results show that the support for initiatives related to speech policies, DEI, and environmental sustainability especially are stronger in the non-white population. In the allocation task experiment, we see that performance in DEI only matters for the non-white population, while a better ranking in free speech only matters for the white population.

Our experimental design focuses on four specific factors that may be important to evaluate universities. However, there may be other factors that are relevant and that are omitted, such as performance in athletics or other aspects of student experience. To avoid introducing too much complexity, we focused on dimensions that had featured prominently in recent public discussions, but future work could examine the relevance of a broader set of factors.

Our findings show there exists a broad consensus on universities having a role in society beyond their core mission of education and research. A wide range of initiatives are supported across the board. Views are, however, split on whether they should engage in DEI initiatives and political engagement. These divisions are not only reflective of typical ideological differences but appear even more accentuated in the current political climate, with contrasts between Harris and Trump voters that are larger than differences along the liberal-conservative line. This polarization is potentially shaped by the political and social context at the time of data collection, which took place in December 2024, after the election of President Trump, but before he took office. At the time, universities had been prominently featured in the U.S. media for their management of student protests in relation to the Israel-Gaza war. There was uncertainty on the actions that the Trump administration would take, but President Trump had publicly indicated his discontent with DEI initiatives and the Republican party had publicly condemned universities for defending the protests on the grounds of free speech. Conservatives and liberals had taken different stances on DEI initiatives, free speech policies and climate sustainability, which could have clearly affected the respondents’ views.

Despite persistent gender and racial disparities in society and in academia (*19*–*21*), our results show a substantial resistance to DEI efforts from both men and conservatives, who go as far as penalizing universities that perform well in this area, and are willing to sacrifice academic performance to reward universities that engage less in DEI. In contrast, liberals see a trade-off between efforts to support DEI initiatives and academic performance. They value academic performance as much as conservatives do, but also value DEI initiatives and are willing to trade-off the two.

It is noteworthy though, that despite these divisions, there is in fact broad consensus on a wide range of initiatives. Health and well-being initiatives, in particular, are perceived very favorably by a large majority. In light of the escalating mental health challenges faced by university students (*22*, *23*), many of whom either refrain from seeking help due to stigma or are unable to access adequate support (*24*), universities are uniquely positioned to take the lead in fostering well-being within their communities. By doing so, they can take on a role that transcends ideological divides.

## MATERIALS AND METHODS

### Experimental design

The objectives of the study were to present evidence on the general public’s views on the role of universities in society, beyond their core missions of education and research. We collected data through a survey among a representative sample of 2218 U.S. citizens via the Prolific platform. The survey was conducted via the survey platform Qualtrics. The experimental design and analyses were preregistered in the American Economic Association Registry for Randomized Controlled Trials (AEARCTR-0015058) on 20 December 2024. The study protocol was granted an exemption from the Institutional Review Board for Human Participants of Cornell University (protocol number IRB0149286).

We requested that this sample would be balanced in age, gender, and political affiliation. We excluded respondents who did not finish the survey. Among them, 1647 respondents completed a survey focused on universities, while 571 responded to a survey about corporations. After excluding respondents who failed attention checks, the final sample consisted of 2089 respondents. Balancing tests confirm that the two samples are balanced across all relevant demographic characteristics (see Supplementary Materials table S15).

The full survey is included in the Supplementary Materials, text S1. The first part of the survey gauges the extent to which respondents agree that major universities or for-profit major corporations should engage in various initiatives beyond their core missions—research and education for universities and core business operations for corporations. Responses were captured on a four-point scale ranging from “definitely should engage,” “probably should engage,” “probably should not engage,” to “definitely should not engage.”

The initiatives included DEI, environmental sustainability, political engagement, speech and expression policies, traditional values, health and well-being programs, global perspectives, free speech and open dialogue, patriotism, and veterans’ initiatives. We provided an explicit description of what was meant by each initiative to limit the extent to which differences in interpretation might affect evaluations (see the Supplementary Materials, text S1, for the description of all initiatives as they were shown to respondents). We also prompted ChatGPT 4.0 about possible interpretations and associations of the initiatives listed using the prompt, “When asked about different initiatives Universities could engage in, what policies do you think respondents could associate with the following general labels:1) Diversity and Inclusion Initiatives, 2) Environmental Sustainability Commitments, 3) Political Engagement, 4) Speech and Expression Policies, 5) Traditional Values, 6) Health and Well-being Programs, 7) Global Perspective, 8) Free Speech and Open Dialogue, 9) Patriotism, 10) Veterans” (see text S2 for the full response). In general, our brief description corresponds well to what is predicted to come to mind to an average person.

Respondents were then asked to gauge the current engagement of universities and corporations for each of the initiatives (options: “too little,” “exactly enough,” “more than enough,” and “do not know”) and if their answers also applied to smaller universities/corporations. Last, the survey also included questions on who should be pushing for social change, and if universities/corporations should take a stance on and allocate resources to different issues (geopolitical conflicts, climate change and environmental issues, DEI, and domestic policies).

Respondents who completed the university-focused survey were then introduced to an experimental paradigm, designed to elicit their views in an incentivized manner. They were presented with information about two existing universities’ ranks in the most recent official rankings across four dimensions (academic performance, environmental sustainability, DEI, and free speech) relative to other universities in the U.S. and given the source for each ranking but not the names of the universities. We chose these dimensions as they have featured prominently in recent public discussions. For academic performance, we used the Shanghai ranking, which is based on several academic and research performance indicators. For environmental sustainability, we used a ranking based on several indicators regarding the sustainable development goals by QS World University Rankings. For DEI, we used a ranking based on several indicators such as racial, gender, and ethnic diversity by a combination of QS World University Rankings and the Social Mobility Index. For free speech, we used a ranking based on several indicators regarding free expression by FIRE Free Speech Rankings.

The respondents were informed that the universities had similar financial endowments [ranging from $560 to $930 million according to the Social Mobility Index website (https://socialmobilityindex.org), which is one of the most recent sources while also providing numbers for a wide range of U.S. universities.].

Each respondent was asked to make five allocation decisions, selected from 22 possible combinations of universities’ pairs (see example screen in Supplementary Materials, text S1). Respondents were informed that 50 out of the approximately 1500 respondents in the study would be randomly chosen, with one decision randomly chosen and implemented for each. Respondents were further told that, consequently, each university could receive up to $1,500 from this study. This approach, consistent with standard practices in the economics discipline, ensured the task was incentivized and mitigates potential social desirability biases in answers (*18*). Moreover, the allocation task forces trade-offs between the different dimensions and thereby helps us identify the relative importance that respondents place on each dimension.

### Statistical analysis

The analysis presented was preregistered. Figures 1 and 2 report raw means, and differences in raw means are tested using *t* tests. Figure 3 presents an OLS analysis of the amount donated to a university (labeled as “University A”) as part of a choice between two universities. The amount is measured in USD and is a number between 0 and 30. The independent variables are dummies indicating if, relative to the other university included in the choice (labeled as “University B”), University A is ranked higher in academic performance, environmental sustainability, DEI, and free speech. The SEs are clustered at the participant level.

We analyze responses averaged over the full sample and over subgroups defined by gender (men versus women), education (no college versus college), and political orientation (conservative versus liberal). The variable female is coded as 1 for respondents identifying as female, 0 for male. college degree is 1 for those with a Bachelor’s, Master’s, or Doctorate, and 0 for those with primary, secondary, or postsecondary education. Conservative, Liberal, and other political are derived from questions on political orientation (1 = “strongly conservative” to 7 = “strongly liberal”) and political identity (Democrat/Republican/Independent/prefer not to answer): Respondents rating 1 to 3 or identifying as Republican are classified as conservative; 5 to 7 or Democrat as liberal. Those meeting both criteria (*n* = 272) were excluded, as this overlap was unanticipated in the preregistration. Including them in both groups yields similar results (available upon request). Other political includes all remaining respondents. The construction of all variables included in the analysis is described in the Supplementary Materials, text S3.

## Supplementary Materials

### This PDF file includes:

Text S1. Survey and Instructions for Respondents Text S2. What comes to mind? Examples of policies associated with initiatives using ChatGPT 4.0 Text S3. Full description of all variables Tables S1 to S20

## References

[1] H. Kaur, N. Yuchtman, Protests on campus: The political economy of universities and social movements. Comp. Econ. Studies 66, 621–638 (2024).

[2] P. Carrese, Civic preparation of American youth: Reflective patriotism and our constitutional democracy. Ann. Am. Acad. Pol. Soc. Sci. 705, 39–52 (2023).

[3] S. Kristinsson, Constructing universities for democracy. Stud. Philos. Educ. 42, 181–200 (2023).

[4] R. J. Daniels, G. Shreve, P. Spector, *What universities owe democracy*, vol. 336 (Johns Hopkins University Press Baltimore, MD) (2021).

[5] United States Census Bureau, Educational Attainment in the United States: 2022, https://www2.census.gov/programs-surveys/demo/tables/educational-attainment/2022/cps-detailed-tables/table-1-1.xlsx, February 2023.

[6] G. Trencher, M. Yarime, K. B. McCormick, C. N. Doll, S. B. Kraines, Beyond the third mission: Exploring the emerging university function of co-creation for sustainability. Sci. Public Policy 41, 151–179 (2014).

[7] G. Trencher, X. Bai, J. Evans, K. McCormick, M. Yarime, University partnerships for co-designing and co-producing urban sustainability. Glob. Environ. Chang. 28, 153–165 (2014).

[8] D. Suárez, P. Bromley, Professionalizing a global social movement: Universities and human rights. Am. J. Educ. 118, 253–280 (2012).

[9] W. L. Filho, S. Weissenberger, J. M. Luetz, J. Sierra, I. S. Rampasso, A. Sharifi, R. Anholon, J. H. P. P. Eustachio, M. Kovaleva, Towards a greater engagement of universities in addressing climate change challenges. Sci. Rep. 13, 19030 (2023). 37923772 10.1038/s41598-023-45866-x PMC10624841

[10] W. L. Filho, J. Sierra, E. Price, J. H. P. P. Eustachio, A. Novikau, M. Kirrane, M. A. P. Dinis, A. L. Salvia, The role of universities in accelerating the sustainable development goals in Europe. Sci. Rep. 14, 15464 (2024). 38965303 10.1038/s41598-024-65820-9 PMC11224266

[11] D. Bok, *Beyond the ivory tower: Social responsibilities of the modern university* (Harvard University Press) (1982).

[12] W. Minter, E. John, I. M. Thompason, *Colleges and Universities as Agents of Social Change* (ERIC) (1968).

[13] J. Emerson, Continuing the work of DEI, no matter what your company calls it. Harv. Bus. Rev. , (2024).

[14] M. Kitzmueller, J. Shimshack, Economic perspectives on corporate social responsibility. J. Econ. Lit. 50, 51–84 (2012).

[15] A. G. Scherer, G. Palazzo, Toward a political conception of corporate responsibility: Business and society seen from a Habermasian perspective. Acad. Manag. Rev. 32, 1096–1120 (2007).

[16] J. Bohnen, *Why Businesses Need a Political Stance. In: Corporate Political Responsibility* (Springer, Berlin, Heidelberg), pp. 85–115 (2021).

[17] D. Baldassarri, A. Gelman, Partisans without constraint: Political polarization and trends in American public opinion. Am. J. Sociol. 114, 408–446 (2008). 10.2139/ssrn.1010098 PMC4056259 24932012

[18] G. Charness, U. Gneezy, B. Halladay, Experimental methods: Pay one or pay all. J. Econ. Behav. Organ. 131, 141–150 (2016).

[19] E. P. Bettinger, B. T. Long, Do faculty serve as role models? The impact of instructor gender on female students. Am. Ec. Rev. 95, 152–157 (2005).

[20] A. Clauset, S. Arbesman, D. B. Larremore, Systematic inequality and hierarchy in faculty hiring networks. Sci. Adv. 1, e1400005 (2015). 26601125 10.1126/sciadv.1400005 PMC4644075

[21] K. Spoon, N. LaBerge, K. H. Wapman, S. Zhang, A. C. Morgan, M. Galesic, B. K. Fosdick, D. B. Larremore, A. Clauset, Gender and retention patterns among U.S. faculty. Sci. Adv. 9, eadi2205 (2023). 37862417 10.1126/sciadv.adi2205 PMC10588949

[22] R. P. Auerbach, J. Alonso, W. G. Axinn, P. Cuijpers, D. D. Ebert, J. G. Green, I. Hwang, R. C. Kessler, H. Liu, P. Mortier, M. K. Nock, S. Pinder-Amaker, N. A. Sampson, S. Aguilar-Gaxiola, A. Al-Hamzawi, L. H. Andrade, C. Benjet, J. M. Caldas-de-Almeida, K. Demyttenaere, S. Florescu, G. de Girolamo, O. Gureje, J. M. Haro, E. G. Karam, A. Kiejna, V. Kovess-Masfety, S. Lee, J. J. McGrath, S. O’Neill, B. E. Pennell, K. Scott, M. TenHave, Y. Torres, A. M. Zaslavsky, Z. Zarkov, R. Bruffaerts, Mental disorders among college students in the World Health Organization world mental health surveys. Psychol. Med. 46, 2955–2970 (2016). 27484622 10.1017/S0033291716001665 PMC5129654

[23] R. P. Auerbach, P. Mortier, R. Bruffaerts, J. Alonso, C. Benjet, P. Cuijpers, K. Demyttenaere, D. D. Ebert, J. G. Green, P. Hasking, E. Murray, M. K. Nock, S. Pinder-Amaker, N. A. Sampson, D. J. Stein, G. Vilagut, A. M. Zasla`ky, R. C. Kessler, WHO world mental health surveys international college student project: Prevalence and distribution of mental disorders. J. Abnorm. Psychol. 127, 623–638 (2018). 30211576 10.1037/abn0000362 PMC6193834

[24] M. Acampora, F. Capozza, V. Moghani, Mental Health Literacy, Beliefs and Demand for Mental Health Support among University Students, Available at SSRN: https://ssrn.com/abstract=4261487 (2022).

